# Prediction Models for Frailty in People Living With HIV: Protocol for a Systematic Review and Meta-Analysis of Prognostic Models

**DOI:** 10.2196/89842

**Published:** 2026-05-26

**Authors:** Yeye Hu, Meilian Xie, Zhiyun Zhang

**Affiliations:** 1 Nursing Management Department, Beijing Ditan Hospital, Capital Medical University, No.8, Jingshun East Street, Chaoyang District, Beijing, 100015, China, 86 13693581102; 2 Nursing Management Department, Capital Center for Children's Health, Capital Medical University, Capital Institute of Pediatrics, Beijing, China

**Keywords:** HIV, frailty, prediction model, systematic review, meta-analysis, PROBAST, CHARMS

## Abstract

**Background:**

With the advent of antiretroviral therapy, the life expectancy of people living with HIV has increased significantly, leading to a growing prevalence of frailty and its associated adverse outcomes. However, frailty prediction models developed for the general older population may not apply to people living with HIV due to their distinct immunologic, inflammatory, and comorbidity profiles. To the best of our knowledge, no systematic review to date has comprehensively evaluated frailty prediction models specifically developed for people living with HIV.

**Objective:**

This systematic review aims to identify and critically appraise existing prediction models for frailty in people living with HIV, critically appraise their methodological quality, and summarize their predictive performance.

**Methods:**

Research will be located by searching electronic databases, including PubMed, Web of Science, and other major databases. Two independent reviewers will conduct the screening of titles and abstracts, evaluate full texts, and extract data. The extraction process will adhere to the Checklist for Critical Appraisal and Data Extraction for Systematic Reviews of Prediction Modeling Studies (CHARMS) and the Transparent Reporting of a Multivariable Prediction Model for Individual Prognosis Or Diagnosis (TRIPOD) statement. A systematic evaluation of the included studies will be performed to assess their risk of bias and applicability, using the Prediction model Risk Of Bias Assessment Tool (PROBAST). If appropriate, meta-analyses will be used to synthesize quantitative data related to the predictive performance of these models.

**Results:**

This protocol was registered with PROSPERO (CRD420261332573) in June 2025. The systematic literature search was conducted on July 20, 2025, with screening completed in October 2025, identifying five eligible studies; data analysis is scheduled for April 2026, with manuscript submission expected by June 2026.

**Conclusions:**

This systematic review will provide the first comprehensive synthesis of frailty prediction models for people living with HIV. By identifying robust models and methodological gaps, the findings are expected to inform clinical decision-making and guide future model development and validation efforts in HIV care.

## Introduction

### Background

AIDS is an infectious disease caused by HIV, which is transmitted via body fluids and secretions [1]. AIDS has evolved from a fatal diagnosis into a chronic condition, mainly owing to the successful implementation of antiretroviral therapy (ART), which has led to a significant increase in life expectancy for people living with HIV [2]. The Joint United Nations Programme on HIV and AIDS (UNAIDS) data show that 40.8 million people were living with HIV globally in 2024 [3]. The figure will rise as ART enables durable viral suppression and substantially extended life expectancy [4]. The proportion of people living with HIV aged 50 years and older is anticipated to increase from 28% in 2010 to 73% in 2030 [5]. As a result, clinicians and public health practitioners are increasingly confronted not only with the direct management of viral load but also with the prevention and treatment of age-related comorbidities and geriatric syndromes in people living with HIV [6].

Among these emerging concerns, frailty, which is characterized by an elevated susceptibility to stressors and diminished physiological reserves and resilience, has garnered particular attention [7]. As a predictor, frailty is closely associated with a variety of adverse health outcomes, including hospitalization, disability, and even death; its prevalence increases markedly with age [8,9]. Mounting evidence [10-12] indicates that people living with HIV experience both an earlier onset and a more severe manifestation of frailty compared with demographically matched, HIV-negative peers, driven by persistent immune activation, chronic inflammation, accelerated immunosenescence, and ART-related metabolic disturbances (eg, dyslipidemia, insulin resistance). A meta-analysis [13] specifically examining frailty in people living with HIV reported that the overall pooled prevalence of frailty and prefrailty was 10.9% and 47.2%. Furthermore, another study [14] indicates that the prevalence of prefrailty was comparable between people living with HIV aged 50 or older and community-dwelling older adults aged 65 or older. Therefore, given the prognostic significance of frailty for adverse outcomes, its rising burden in PLWH underscores an urgent need for effective screening and early intervention strategies.

A clinical prediction model [15] is a statistical tool that combines multiple predictors—demographic, clinical, laboratory, or imaging variables—to estimate an individual’s risk of having a given condition (diagnostic model) or developing it (prognostic model). This research primarily focuses on prognostic models that predict frailty in people living with HIV in clinical settings. Numerous frailty prediction models exist within geriatric medicine. Two main clinical tools consist of the frailty index (FI) [16] and the frailty phenotype (FP) [17], the FP defines frailty as represented by poor performance in three of five criteria (ie, weight loss, exhaustion, weakness, slowness, lack of activity). In contrast, the FI is the result of the accumulation of various health deficits. However, applying standardized FP or FI directly to people living with HIV for frailty assessment or risk prediction poses significant challenges. These instruments typically do not incorporate key pathophysiological drivers specific to HIV infection. Such as: minimum CD4 count, virus load, chronic immune activation, ART regimens, and common coinfections [18,19]. Besides, the comorbidity spectrum, patterns of functional decline, and psychosocial stressors among people living with HIV differ from those in the general elderly population [20]. The above reasons collectively suggest that frailty prediction models for the general population may lack direct transferability to people living with HIV.

Consequently, there has been a growing research focus over the past decade on developing prediction tools specifically calibrated for people living with HIV [21,22]. However, despite these research advances and the critical need for tailored predictive tools in HIV care, a comprehensive, systematic evaluation synthesizing the methodological rigor, predictive performance, and clinical applicability of existing HIV-specific frailty prediction models remains conspicuously absent from the scientific literature. This critical knowledge gap, confirmed through preliminary searches of major biomedical databases, underscores the necessity and novelty of the proposed systematic review.

### Research Aims

The planned study aims to conduct a systematic review of all available evidence regarding the current frailty models for people living with HIV and to identify which prediction models have been developed, establishing the most effective and best-performing model to predict frailty, while informing clinical decision-making. The specific aims of this systematic review are:

1. To ascertain the existing frailty prediction models for people living with HIV
2. To summarize and compare the current frailty prognostic prediction models and their predictive performance
3. To critically appraise the studies identified for inclusion, particularly the research methodology and reporting methods

## Methods

### Methodology

This present protocol was formulated in adherence to the Preferred Reporting Items for Systematic Review and Meta-Analysis Protocol (PRISMA-P) guidelines [23] and was duly registered with PROSPERO, the international prospective register of systematic reviews.

A systematic review of prognostic prediction modeling studies for people living with HIV with frailty will be rigorously conducted and reported in strict accordance with the best-practice guidelines established by the CHARMS (Checklist for critical Appraisal and data extraction for systematic Reviews of prediction Modelling Studies) framework [24]. Adherence to these internationally recognized standards will be maintained throughout all stages of the review process, encompassing: (1) the formulation of the research question and eligibility criteria; (2) systematic search strategy development and execution; (3) study selection; (4) data extraction; (5) risk of bias and applicability assessment using recommended tools (eg, PROBAST) [25]; and (6) narrative synthesis of findings. This structured approach will ensure methodological rigor, transparency, and reproducibility, providing a robust foundation for understanding the current state of prediction modeling for HIV-associated frailty and identifying key research gaps.

### Eligibility Criteria

The outline of the review data and study selection was defined according to the CHARMS checklist (key items to guide the framing of the review aim, search strategy, and study inclusion and exclusion criteria).

The inclusion criteria are as follows: (1) studies that develop or validate prediction models, whether or not they include external validation; (2) study populations that involve research on HIV or AIDS diagnosed by any of the following recognized guidelines: Chinese guidelines for the diagnosis and treatment of human immunodeficiency virus infection/acquired immunodeficiency syndrome [26], Consolidated Guidelines on HIV Testing Services [27], Laboratory Testing for HIV (2023) (US Centers for Disease Control and Prevention), European AIDS Clinical Society (EACS) version 12.1 [28], UK Testing Guidelines [29]; (3) studies on adults older than 18 years; (4) primary outcome measures indicating the future occurrence of frailty, where frailty is defined using established multidimensional frailty assessment instruments such as the FP, FI, Clinical Frailty Scale, Edmonton Frail Scale, FRAIL Scale, or other validated frailty measures; (5) studies that develop or validate statistical models, machine learning algorithms, or clinical risk scores for predicting an individual’s risk of future frailty, regardless of the terminology used (eg, prediction model, risk score, prediction index, clinical prediction rule).

The exclusion criteria are as follows: (1) evaluation of the predictive value of more than one variable but without reporting subgroups or evaluation outcomes; (2) cross-sectional studies, as they are not designed to assess future risk prediction; (3) nonprimary research such as literature reviews, systematic reviews, meta-analyses, protocols, theses, editorial comments, or letters; and (4) studies not available in full text.

There will be no restrictions on year or language. In instances where multiple studies report results from the same cohort concerning a specific outcome measure, the data from the study that encompasses the largest patient population will be selected for analysis. Alternatively, if the studies involved an equal number of patients, the data from the earliest published study will be used.

### Information Sources

The main databases to be searched are SinoMed, CNKI, Wanfang, VIP, PubMed, Web of Science, Scopus, Cochrane Library; grey database: ProQuest from database creation to present.

### Search Strategy

Subject indexing terms will include a combination of the following three aspects of the PICOS (Population, Intervention, Comparator, Outcome, Study design) system search construct: #1 Population search AND #2 Index search AND #3 Outcomes search.

1. Population search: Terms related to HIV/AIDS, eg, HIV, AIDS, human immunodeficiency virus, HIV infections
2. Index search: Terms related to prediction models, eg, risk assessment, risk prediction, prediction model, prognostic model, risk score, nomogram
3. Outcomes search: Terms related to frailty, eg, frailty, frail, frailty phenotype, frailty index

All model development studies will be citation-searching to identify potentially relevant external validation studies. Subsequently, a comprehensive review of all retrieved studies will be performed to ascertain their suitability for inclusion in the analysis. References identified by the search strategy will be entered into EndNote software to screen the selected articles.

### Study Selection

Based on the search strategy, after removing the duplicated articles, two authors (YH and MX) will independently screen the titles and abstracts. The search results will then be screened a second time, in duplicate. Potential disagreements regarding the inclusion of an article will be resolved through a discussion, but in case of differences, a third researcher (ZZ) decides whether to include an article. If insufficient information is available in the published report to determine eligibility, we will attempt to contact the corresponding authors to obtain the necessary details. If the information cannot be obtained, the study will be recorded as having missing data in the data extraction form rather than automatically excluded; the absence of such information will be noted as a limitation in the discussion. All studies meeting the inclusion criteria will be included regardless of their risk of bias, as assessed using the PROBAST tool. To evaluate the impact of methodological quality on the overall findings, sensitivity analyses will be conducted by excluding studies at high risk of bias, thereby assessing the robustness of the pooled estimates.

### Data Collection Process

Data extraction will be conducted independently by two members (YH and MX) of the study team using a standardized and piloted data extraction with the CHARMS.

.A checklist of relevant items (for purposes of description or assessment of risk of bias or applicability) will be used. Moreover, the data items in the checklist will adapt to the specific clinical question, for instance, aims; data source; participants; stakeholders; algorithms; predicted outcomes; potential predictors; sample size; missing data; model development; model performance, including properties of discrimination with confidence intervals, calibration, classification, and overall performance; final multivariable models; interpretation of presented models; and model evaluation.

### Critical Appraisal

PROBAST will be used to analyze the methodological quality and relevance of participants, predictors, and outcomes from each included study to the review topic in a systematic assessment. With a total of 20 signaling questions, this instrument comprises four domains: participants, predictors, outcomes, and analysis. Domains were scored as “high,” “low,” or “unclear” risk of bias. Two reviewers (YH and MX) will independently apply the tool to rate the risk of bias and applicability of each included study. The kappa coefficient for inter-rater reliability should be over 0.8. Any disagreement will be resolved by discussion.

### Evidence Synthesis

The initial approach will be to use a narrative synthesis method to systematically describe the characteristics of the included studies and the quantitative data obtained for descriptive analysis in detail [30]. The final results will be presented in a table alongside the original studies.

### Meta-Analysis

Where feasible, this study will use a random-effects meta-analysis model to conduct a comprehensive analysis of relevant performance metrics. These metrics will encompass the model’s discriminatory ability, as measured by consistency statistics or the area under the curve, and its calibration ability, as indicated by the total ratio of observed to expected events and the calibration slope. This will allow us to infer the predictive model’s average performance across the included studies. To assess heterogeneity among the studies and determine the 95% confidence interval for the average performance, the study will apply the restricted maximum likelihood method and the Hartung-Knapp-Sidik-Jonkman method separately [24].

To ascertain the robustness of the findings, sensitivity analyses will be conducted, wherein studies deemed to have a significant or uncertain risk of bias will be excluded. Potential sources of considerable between-effects heterogeneity will be investigated by conducting a meta-regression analysis (*P*<.05). If possible, the subgroup analysis will be based on:

1. Risk factors: biomarkers or psychosocial factors
2. Outcomes: primary outcomes (frailty)
3. frailty types: physical frailty, cognitive frailty, social frailty (if reported and clearly distinguished in the included studies)
4. Modeling techniques: machine learning or non-machine learning
5. Follow-up duration
6. Region: based on the Organization for Economic Co-operation and Development classification, that is, low/middle-income and high-income countries

The process of meta-analysis will be carried out using the *metareg* module in Stata 15.0, following the recommendations set forth by the Meta-analysis of Observational Studies in Epidemiology (MOOSE) group [31].

However, given the anticipated heterogeneity in study populations, frailty definitions, and prediction models, as well as the potentially limited number of included studies (as suggested by our preliminary search identifying five eligible studies), a comprehensive quantitative synthesis may not be feasible. If quantitative synthesis is not feasible due to insufficient data or excessive heterogeneity, we will provide a narrative synthesis of the included studies, summarizing their characteristics, predictive performance, and methodological quality in tabular format, following the guidance for systematic reviews without meta-analysis (SWiM).

### Ethical Considerations

Ethical approval is not applicable to this study as it is a systematic review of published literature.

## Results

**Figure 1.**
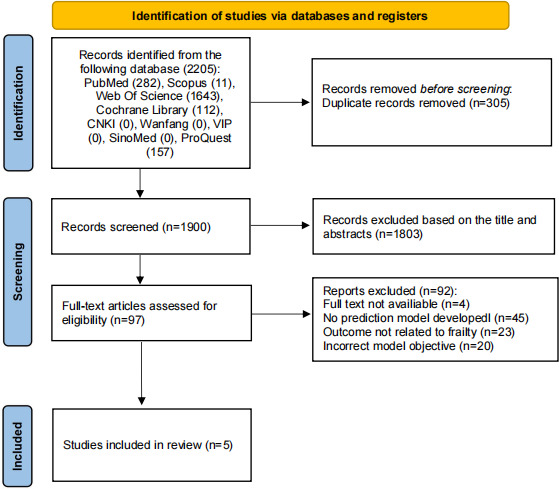
PRISMA (Preferred Reporting Items for Systematic Reviews and Meta-Analyses) flow diagram of the study selection process. A total of 2205 records were identified from the following databases: PubMed (282), Scopus (11), Web of Science (1643), Cochrane Library (112), ProQuest (157), and four Chinese databases (CNKI, Wanfang, VIP, and SinoMed) with zero records. After removing 305 duplicate records, 1900 records were screened based on titles and abstracts, of which 1803 were excluded. The remaining 97 full-text articles were assessed for eligibility, and 92 were excluded for the following reasons: full text not available (n=4), no prediction model developed (n=45), outcome not related to frailty (n=23), and incorrect model objective (n=20). Ultimately, 5 studies met the inclusion criteria for the systematic review. Adapted from the Preferred Reporting Items for Systematic Reviews and Meta-Analyses (PRISMA) 2020 statement.

This protocol was registered with PROSPERO (CRD420261332573) in June 2025. The systematic literature search was conducted on July 20, 2025, across major databases including PubMed, Web of Science, Scopus, Cochrane Library, grey database: ProQuest, and four Chinese databases (SinoMed, CNKI, Wanfang, VIP). Title and abstract screening commenced in September 2025 and was completed in October 2025, with a total of five studies meeting the inclusion criteria after full-text review. Data extraction and quality appraisal using the PROBAST tool are planned for the next phase, with data analysis scheduled for April 2026 and manuscript submission expected by June 2026. The study selection process is summarized in Figure 1.

## Discussion

### Principal Findings

This planned study will be the first systematic review to evaluate prognostic prediction models for frailty in people living with HIV. Adhering to CHARMS, TRIPOD, and PROBAST, and where feasible conducting meta-analyses, this review aims to provide a rigorous synthesis of existing evidence. This review is expected to reveal significant differences among models regarding variable selection (eg, CD4 count, inflammatory markers, and traditional physical fitness indicators) and definitions of frailty (eg, FP, FI). The observed heterogeneity suggests that there is an absence of consensus within the field regarding core predictors and standardized outcome definitions. This emphasizes the pressing need for harmonization in future research endeavors. Moreover, the identification of HIV-specific factors (eg, ART regimens, immune status) as being underrepresented in existing models indicates that current approaches may not fully capture the unique risk profile of people living with HIV, thereby underscoring the necessity for the development of tailored models.

These observations have several important implications for both clinical practice and future research. First, identifying the most robust and well-validated models will provide clinicians with evidence-based tools for early identification of people living with HIV at risk of frailty, enabling timely interventions and personalized management. Second, the review will inform future research by highlighting methodological strengths and weaknesses of existing studies, emphasizing the necessity of external validation, particularly multicenter validation in low- and middle-income populations to enhance generalizability and clinical utility. Based on these insights, we will propose recommendations for standardized frailty measurement, unified assessment processes, and integration of dynamic data from electronic health records.

However, several limitations should be acknowledged. Despite comprehensive searches across major databases, we may miss relevant studies published in non-indexed journals or non-English languages; practical constraints in accessing and translating such studies could introduce language bias. Moreover, the small number of eligible studies identified in our preliminary search (n=5) suggests that the evidence base may be limited, potentially constraining the strength and generalizability of our conclusions. The anticipated heterogeneity across study populations, frailty definitions, and modeling techniques may restrict the feasibility of quantitative synthesis. In addition, variability in model reporting—such as incomplete presentation of discrimination (eg, C-statistics) or calibration metrics—could further limit the potential for meta-analysis and may introduce reporting bias. If quantitative synthesis is not feasible, we will provide a narrative synthesis following the SWiM guidelines, as outlined in the Methods section. Finally, the validity of our findings will depend on the quality and completeness of reporting in the included primary studies.

Despite these limitations, this review has the potential to change both clinical practice and future research directions. By systematically synthesizing available evidence, it will provide a clear picture of the current state of frailty prediction modeling in HIV care, highlighting critical gaps such as the lack of externally validated models and the underrepresentation of certain populations. In clinical practice, the identification of well-performing models will support the integration of frailty risk assessment into routine HIV care, potentially shifting the focus from reactive management of complications to proactive, personalized prevention strategies. Ultimately, this work aims to contribute to improved quality of life and health outcomes for the aging HIV population.

### Conclusion

This research aims to identify the independent predictors of frailty in people living with HIV. By developing and validating prediction models, the findings from this study will inform clinical risk assessment, support informed decision-making, and facilitate personalized treatment approaches. Moving forward, it is essential to gather ample data to confirm the current frailty measures and evaluate their clinical effectiveness in PLWH.

## Abbreviations

ART: antiretroviral therapy
CHARMS: Checklist for critical Appraisal and data extraction for systematic Reviews of prediction Modelling Studies
FI: frailty index
FP: frailty phenotype
MOOSE: Meta-analysis of Observational Studies in Epidemiology
PRISMA-P: Preferred Reporting Items for Systematic Review and Meta-Analysis Protocol
PROBAST: Prediction model Risk Of Bias ASsessment Tool
SWiM: systematic reviews without meta-analysis
TRIPOD: Transparent Reporting of a multivariable prediction model for Individual Prognosis Or Diagnosis
UNAIDS: Joint United Nations Programme on HIV and AIDS
